# Patterns of care and outcomes in immigrants with non-small cell lung cancer. A population-based study (Sweden)

**DOI:** 10.1371/journal.pone.0278706

**Published:** 2022-12-15

**Authors:** Linda Willén, Anders Berglund, Stefan Bergström, Johan Isaksson, Michael Bergqvist, Gunnar Wagenius, Mats Lambe

**Affiliations:** 1 Center for Research and Development, Uppsala University/Region Gävleborg, Gävle, Sweden; 2 Department of Radiation Sciences, Oncology, Umeå University, Umeå, Sweden; 3 Department of Oncology, Gävle Hospital, Gävle, Sweden; 4 EpiStat AB, Uppsala, Sweden; 5 Department of Pulmonary Medicine, Gävle Hospital, Gävle, Sweden; 6 Department of Immunology, Genetics and Pathology, Uppsala University, Uppsala, Sweden; 7 Section of Oncology, Uppsala University Hospital, Uppsala, Sweden; 8 Division of Oncology, Department of Clinical Science Intervention and Technology, Karolinska Institutet, Stockholm, Sweden; 9 Department of Medical Epidemiology and Biostatistics, Karolinska Institutet, Stockholm, Sweden; 10 Regional Cancer Center Central Sweden, Uppsala, Sweden; The Armed Forces Goyang Hospital, REPUBLIC OF KOREA

## Abstract

**Objectives:**

While studies have found lower cancer risks and better cancer survival in immigrant populations, it is debated whether cancer care is offered on equal terms to all residents regardless of background. Our aim was to study patterns of care and outcomes in immigrants in a country with a tax-financed universal health care system.

**Material and methods:**

We used a population-based database to compare clinical presentation, management and mortality between Swedish-born and immigrant patients with non-small cell lung cancer (NSCLC). Analyses were adjusted for potential confounders.

**Results:**

We identified 40,075 patients diagnosed with NSCLC of which 84% were born in Sweden, 7% in Nordic and 9% in Non-Nordic countries. Non-Nordic immigrants were to a higher extent male, smokers, younger at diagnosis, had a better performance status and a higher educational level. No differences were seen regarding comorbidity burden or stage at diagnosis. Non-Nordic immigrants more often underwent positron emission tomography (PET) (aHR 1.32; 95% CI 1.19–1.45) and were more often discussed in a multidisciplinary team setting (aHR 1.30; 95% CI 1.17–1.44). There were no differences in treatment modalities following adjustment for age, with the exception of concurrent chemoradiotherapy in stage IIIA disease which was more common in Non-Nordic immigrants (aOR 1.34; 95% CI 1.03–1.74). Both overall and cause specific survival in non-metastatic disease were higher among Non-Nordic immigrants. Overall mortality in stage I-II: HR 0.81; 95% CI 0.73–0.90 and stage IIIA: HR 0.75; 95% CI 0.65–0.86. Following full adjustments, cause-specific mortality in stage I-II was aHR 0.86, 95% CI 0.75–0.98.

**Conclusion:**

Taken together, only minor differences in management and outcomes were observed between Swedish-born and immigrant patients. We conclude that lung cancer care is offered on equal terms. If anything, outcomes were better in Non-Nordic immigrants with early stage NSCLC.

## 1. Introduction

It is debated whether countries with tax-funded national health care systems provide care to all residents on equal terms. Also in settings with universal health care, sociodemographic factors such as educational level, income, age, sex and migration status have been shown to be associated with cancer incidence and outcomes, including lung cancer [1–5]. Differences in age, life-style, language skills and ability to navigate the health care system have been attributed to health differences between native and immigrant populations [6, 7].

Several studies have found evidence of lower cancer risks and better cancer survival in immigrant populations [8–10], findings which are likely to reflect the ‘healthy migrant effect’, i.e. immigrants being on average healthier, younger and better educated compared both to the populations of their country of origin and host country [11, 12]. This health selection phenomena appears to be more pronounced in immigrants relocating for education and labor purposes compared to refugees and family reunification immigrants, but with differences diminishing over time [13, 14]. A demographic shift due to low birth rates and increasing immigration numbers has increased the proportion of foreign-born residents in Sweden from about 12% in 2006 to about 20% in 2019 [7, 15].

We used information in a population-based lung cancer research database to compare clinical presentation, diagnostic intensity, treatment and outcomes between foreign born and native born patients diagnosed with non-small cell lung cancer (NSCLC) in Sweden, a country with a national health care system aiming to offer care on equal terms to all residents.

## 2. Materials and methods

### 2.1 Data collection

For the purpose of this population-based cohort study we used data from Lung Cancer Data Base Sweden, a research database generated by record linkages between the National Lung Cancer Register and several other Swedish population-based registers. The setup of Lung Cancer Data Base Sweden has previously been described in detail [4, 16, 17].

In short, the National Lung Cancer Register contains individual level information on smoking history, performance status, diagnostics, tumor characteristics (tumor stage and histopathology) and first line treatment. Smoking history is recorded based on self-report and categorized as smoker (current smoker), former smoker (no smoking during the last year) and non-smoker (never smoked on a regular basis). Performance status (PS) is based on the World Health Organization (WHO) performance score as assessed by the treating physician [18].

By means of record linkage, information in the National Lung Cancer Register was supplemented with individual level information available in the National Patient Register, the Swedish Cancer Register, the social database Longitudinal Integration Database for Health Insurance and Labour Market Studies and the Cause of Death Register.

The National Patient Register contains information on diagnosis made in hospital in- and outpatient settings and was used to estimate the Charlson Comorbidity Index (CCI) [19] categorized into three groups; no (CCI 0), mild (CCI 1–2), and severe comorbidity (CCI ≥ 3). The Swedish Cancer Register, to which reporting is mandated by law, includes data on all cancer diagnoses as reported by clinicians and pathologists.

The continuously updated Longitudinal Integration Database for Health and Insurance and Labour Market Studies database includes information on several different indicators of socioeconomic position [20]. For the purpose of the present study socioeconomic status was assessed based on highest achieved educational level collapsed into three groups: low ≤9 years of schooling, middle 10–12 years and high ≥13 years, corresponding to mandatory school, high school and post-high school (college and university).

Date and cause of death was available in the Cause of Death Register that includes data on main and contributing causes of death [21]. The completeness of the Cause of Death Register is high and is commonly used to assess cause-specific survival [22].

The study population was divided into three groups according to region of birth; “Sweden”, “Nordic” including residents born in the other Nordic countries (Denmark, Finland, Iceland and Norway), and “Non-Nordic” including all non-Nordic countries of origin. Because of small numbers, further subgrouping of Non-Nordic immigrants based on country or geographic region of origin was not possible.

### 2.2 Statistical methods

Demographic characteristics, clinical characteristics and patterns of management (diagnostic and treatment intensity) in all patients diagnosed with non-small cell lung cancer in Sweden 2002–2016 are presented. Comparison between the groups were made using the Chi square test. Univariable and multivariable logistic regression models were used to examine possible differences in management, including treatment modalities, based on region of birth. Data were stratified by stage at diagnosis and with analyses restricted to patients with PS 0–2 adjusted for age, sex, educational level, smoking history, stage at diagnosis, CCI, PS, histopathology, health care region in Sweden and year of diagnosis. The selection of patients with PS 0–2 was made to include patients eligible for oncological treatment. In a final step, cause-specific and overall mortality were compared between region of birth using the Kaplan-Meier method and Cox regression models with Hazard Ratios and 95% confidence intervals. Missing data were excluded in all the analyses.

Survival time was calculated as the time interval between the date of lung cancer diagnosis and date of death, and patient were censored at emigration or end of follow-up (December 31, 2016), whichever came first. In the cause-specific analyses, death due other causes were censored. All tests were two-sided, and a 5% level was considered statistically significant. Statistical analyses were performed using R version 3.5.0.

### 2.3 Research ethics

The study was approved by the Research Ethics Board in Stockholm (2016/1137-32; 2017/2026-32; 2017-445-32).

## 3. Results

### 3.1 Demographics and clinical characteristics

We identified a total of 53,359 patients diagnosed with lung cancer between 2002 and 2016. Following exclusion of histopathological subtypes other than non-small cell lung cancer (n = 12,490) and missing data on educational level (n = 794), the final study population consisted of 40,075 men and women with non-small cell lung cancer. Demographic and clinical characteristics are presented in Table 1.

**Table 1:** Demographic and clinical characteristics.

	Region of birth								
	Sweden		Nordic		Non-Nordic			Total	
	n	%	n	%	n	%	p-value	n	%
**All cases**	33496	100	2968	100	3611	100		40075	100
**Sex**							<0.001		
Male	16820	50.2	1597	53.8	2393	66.3		20810	51.9
Female	16676	49.8	1371	46.2	1218	33.7		19265	48.1
**Age at diagnosis**							<0.001		
0–59	4615	13.8	454	15.3	1057	29.3		6126	15.3
60–69	11396	34.0	1065	35.9	1236	34.2		13697	34.2
70–79	12369	36.9	1116	37.6	967	26.8		14452	36.1
80–89	4932	14.7	330	11.1	339	9.4		5601	14
90+	184	0.5	3	0.1	12	0.3		199	0.5
**Educational level**							<0.001		
Low	14686	43.8	1532	51.6	1296	35.9		17514	43.7
Middle	13585	40.6	1161	39.1	1515	42.0		16261	40.6
High	5225	15.6	275	9.3	800	22.2		6300	15.7
**Charlson Comorbidity Index**							<0.001		
CCI 0	18254	54.5	1534	51.7	2038	56.4		21826	54.5
CCI 1–2	9953	29.7	944	31.8	971	26.9		11868	29.6
CCI 3+	5289	15.8	490	16.5	602	16.7		6381	15.9
**Smoking history**							<0.001		
Current	14188	42.4	1523	51.3	1750	48.5		17461	43.6
Former	14920	44.5	1214	40.9	1322	36.6		17456	43.6
Never	3762	11.2	171	5.8	463	12.8		4396	11.0
Missing	626	1.9	60	2.0	76	2.1		762	1.8
**WHO performance status**							<0.001		
0	7319	21.9	578	19.5	966	26.8		8863	22.1
1	12653	37.8	1141	38.4	1375	38.1		15169	37.9
2	6981	20.8	621	20.9	677	18.7		8279	20.7
3	3995	11.9	383	12.9	353	9.8		4731	11.8
4	1412	4.2	132	4.4	107	3.0		1651	4.1
Missing	1136	3.4	113	3.9	133	3.6		1382	3.4
**Histopathology**							<0.001		
Adenocarcinoma	19974	59.6	1609	54.2	2138	59.2		23721	59.2
Squamous cell	8461	25.3	892	30.1	953	26.4		10306	25.7
Large cell	4689	14.0	423	14.3	475	13.2		5587	13.9
Adenosquamous	372	1.1	44	1.5	45	1.2		461	1.2
**Stage at diagnosis**							0.085		
IA-IIB	8294	24.8	754	25.4	893	24.7		9941	24.8
IIIA	3094	9.2	286	9.6	385	10.7		3765	9.4
IIIB-IV	21584	64.4	1888	63.6	2295	63.6		25767	64.3
Missing	524	1.6	40	1.4	38	1.0		602	1.5

Demographic and clinical characteristics of patients diagnosed with non-small cell lung cancer in Sweden 2002–2016 by geographic region of birth.

Abbreviations: WHO: World Health Organization

In the population under study, 83.6% were born in Sweden, 7.4% in the other Nordic countries and 9.0% in Non-Nordic countries. There was a marked male predominance in the Non-Nordic cohort (Sweden 50.2%, Nordic 53.8% and Non-Nordic 66.3%). Compared to native born Swedes the prevalence of current smoking was higher in immigrants (Sweden 42.4%, Nordic 51.3% and Non-Nordic 48.5%).

Non-Nordic patients were younger at diagnosis (age 0–59 years: Sweden 13.8%, Nordic 15.3% and Non-Nordic 29.3%), had a better performance status (PS 0: Sweden 21.9%, Nordic 19.5% and Non-Nordic 26.8%) and a higher educational level (post-high school: Sweden 15.6%, Nordic 9.3% and Non-Nordic 22.2%). A histopathology of squamous cell carcinoma was slightly more common in Nordic immigrants (Sweden 25.3%, Nordic 30.1% and Non-Nordic 26.4%). There were no differences in comorbidity burden as assessed by CCI or stage at diagnosis.

### 3.2 Diagnostic intensity and multidisciplinary team assessment

Diagnostic work-up with bronchoscopy, endobronchial ultrasound (EBUS), computed tomography (CT) thorax, ultrasound (US) or CT abdomen, thoracocentesis, thoracoscopy, transthoracal biopsy and CT or MRI brain and, in adenocarcinomas, testing for epidermal growth factor receptor (EGFR) mutation were evenly performed across all groups (Table 2).

**Table 2:** Diagnostic intensity.

	Region of Birth								
	Sweden		Nordic		Non-Nordic		p-value	Total	
	n	%	n	%	n	%		n	%
**Diagnostic intensity**	** **	** **	** **	** **	** **	** **	** **	** **	** **
Bronchoscopy	24622	73.5	2228	75.1	2744	76.0	0.003	29594	73.8
Bronchoscopy EBUS**	2363	10.8	143	7.4	326	12.8	<0.001	2832	10.8
CT Thorax	32689	97.6	2901	97.7	3533	97.8	0.805	39123	97.6
Mediastinoscopy	2109	6.3	195	6.6	263	7.3	0.071	2567	6.4
US/CT upper abdomen	29964	89.5	2629	88.6	3224	89.3	0.225	35817	89.4
Thoracocentesis	4410	13.2	349	11.8	371	10.3	<0.001	5130	12.8
Thoracoscopy	454	1.4	32	1.1	51	1.4	0.420	537	1.3
Transthoracic biopsy	9242	27.6	817	27.5	830	23.0	<0.001	10889	27.2
CT/MRI brain***	5880	24.6	532	25.1	768	27.9	0.001	7180	24.9
Other	7236	21.6	625	21.1	810	22.4	0.541	8671	21.6
PET scan***	10256	42.9	873	41.3	1390	50.5	<0.001	12519	43.5
EGFR test****	6364	55.7	494	54.0	721	54.0	0.369	7579	55.4
**Multidisciplinary team assessment**	21245	63.4	1920	64.7	2533	70.1	<0.001	25698	64.1

Diagnostic intensity and multidisciplinary team assessment in patients diagnosed with non-small cell lung cancer in Sweden 2002–2016 by geographic region of birth.*

*Some patients received multiple diagnostic modalities.

** Data only available for 2008–2016

*** Data only available for 2007–2016

****Data only on adenocarcinomas diagnosed 2010–2016

Abbreviations: EBUS: endobronchial ultrasound; CT: computed tomography; US: Ultrasound; MRI: magnetic resonance imaging; PET: positron emission tomography; EGFR: epidermal growth factor receptor.

Non-Nordic immigrants more often underwent positron emission tomography (PET) (Sweden 42.9%, Nordic 41.3% and Non-Nordic 50.5%) and were more often discussed in a multidisciplinary team setting (MDT) (Sweden 63.4%, Nordic 64.7% and Non-Nordic 70.1%). (Table 2). Following adjustments for age these differences were significant in Non-Nordic immigrants for both PET and MDT (aOR 1.22 (95% CI 1.13–1.33); aOR 1.29 (95% CI 1.18–1.42) and for MDT in Nordic immigrants (aOR 1.12 (95% CI 1.01–1.24). After adjustments for further potentially modifying factors (age, sex, level of education, CCI, stage, year of diagnosis, performance status, smoking history and histopathology) these differences remained (Non-Nordic immigrants PET aOR 1.32 (95% CI 1.19–1.45) and MDT aOR 1.30 (95% CI 1.17–1.44); Nordic immigrants MDT aOR 1.19 (95% CI 1.07–1.33)).

### 3.3 Treatment intensity

First line treatment modalities in patients with PS 0–2 and stage IA-IV NSCLC are presented in Table 3.

**Table 3:** Treatment modality.

	Sweden		Nordic		Non-Nordic		Total	
	n	%	n	%	n	%	n	%
**Stage IA-IIB, WHO PS 0–2**	** **	** **	** **	** **	** **	** **	** **	** **
Surgery	5698	73.0	483	68.8	665	77.8	6846	73.1
Stereotactic radiotherapy**	677	11.7	76	14.6	75	11.5	828	11.9
**Stage IIIA, WHO PS 0–2**								
Surgery	480	17.4	38	15.1	84	24.4	602	17.9
Concurrent chemoradiotherapy**	920	43.4	98	49.7	163	57.2	1181	45.4
Radiotherapy	1231	44.5	108	43.0	146	42.4	1485	44.2
**Stage IIIB-IV, WHO PS 0–2**								
Chemotherapy	12942	80.3	1075	78.9	1436	80.0	15453	80.2
Concurrent chemoradiotherapy**	1008	8.8	80	8.4	160	11.6	1248	9.1
Radiotherapy	2989	18.6	262	19.2	373	20.8	3624	18.8

Treatment modality in patients diagnosed with non-small cell lung cancer in Sweden 2002–2016 with performance status 0–2 by geographic region of birth.*

*Some patients received multiple treatment modalities.

** Data only available for 2007–2016

Abbreviations: WHO PS: World Health Organization Performance Score

#### 3.3.1 Stage IA-IIB

In patients with PS 0–2 and stage IA-IIB disease, surgery was more commonly performed in Non-Nordic immigrants (Sweden 73.0%, Nordic 68.8% and Non-Nordic 77.8%). In unadjusted analyses the likelihood of surgery was almost 30% higher (OR 1.29 (95% CI 1.09–1.52)), but became attenuated following adjustment for age (aOR 0.85 (95% CI 0.71–1.02)). Further adjustments for factors potentially affecting treatment decisions did not materially change this estimate (aOR 0.83 (95% CI 0.67–1.03)) (S1 Table).

Stereotactic radiotherapy (SBRT) was slightly more common in Nordic immigrants (14.6%) compared to Swedish-born (11.7%) and Non-Nordic (11.5%) patients, a difference that was significant after adjustment for age (Nordic: aOR 1.38 (95% CI 1.01–1.89)), but not in a fully adjusted model (Nordic: aOR 1.36 (95% CI 0.94–1.96) (S2 Table).

#### 3.3.2 Stage IIIA

In patients with PS 0–2 and stage IIIA disease, treatment with surgery and concurrent chemoradiotherapy was more common in Non-Nordic immigrants (surgery: Sweden 17.4%, Nordic 15.1% and Non-Nordic 24.4%; concurrent chemoradiotherapy: Sweden 43.4%, Nordic 49.7% and Non-Nordic 57.2%). There were no differences in treatment modalities following adjustment for age, with the exception of concurrent chemoradiotherapy that was more commonly used in Non-Nordic immigrants (aOR 1.34 (95% CI 1.03–1.74). (S3 and S4 Tables). There were no differences in the use of radiotherapy alone.

#### 3.3.3 Stage IIIB-IV

In patients with PS 0–2 and advanced IIIB-IV disease, there were no differences between groups in treatments given.

### 3.4 Overall and cause-specific mortality

**Fig 1:**
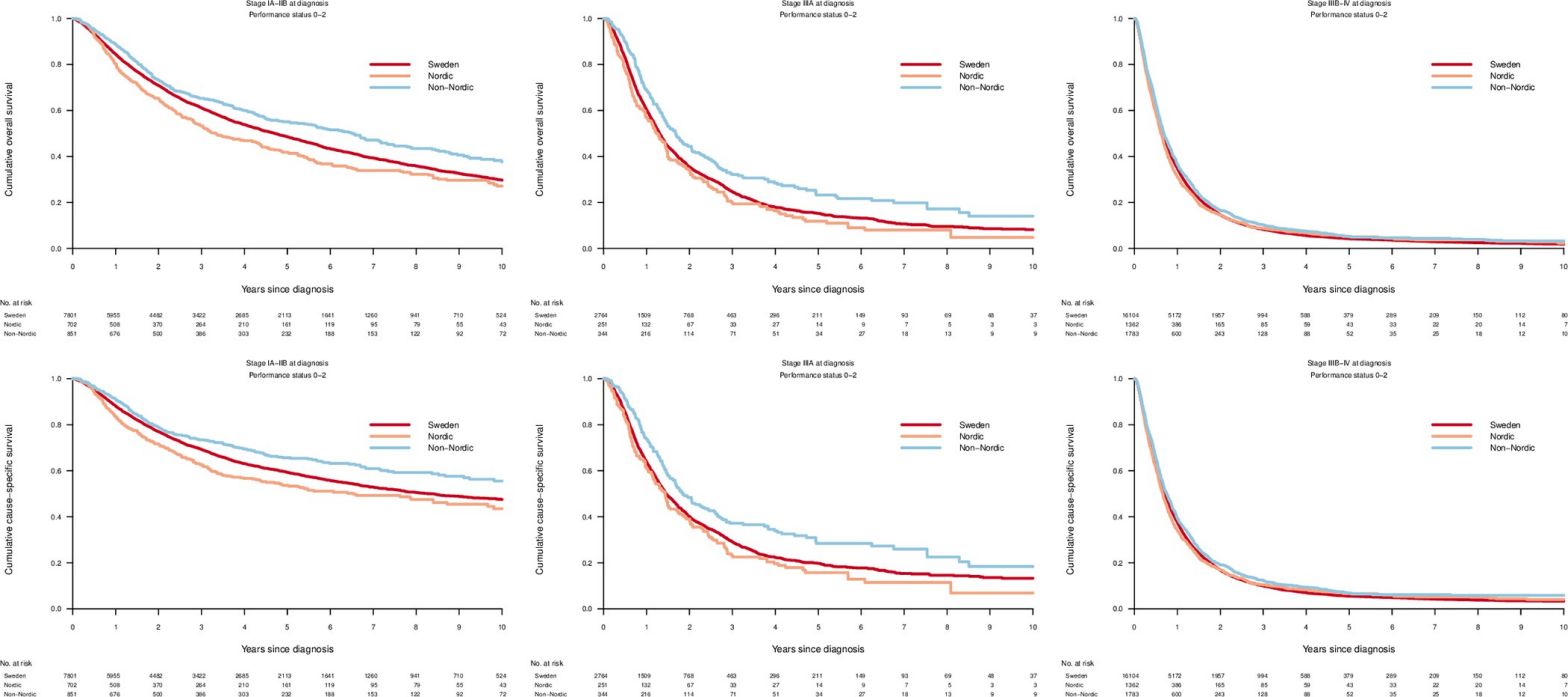
Overall and cause-specific survival. Cumulative overall and cause-specific survival in patients with performance status 0–2 diagnosed with non-small cell lung cancer in Sweden 2002–2016 by geographic region of origin.

In stage IA-IIB and IIIA, but not in advanced stage IIIB-IV disease, overall and cause-specific survival was higher in Non-Nordic immigrants compared to Nordic immigrants and Swedish born patients. (Fig 1). In a fully adjusted model this was reflected in lower overall and cause-specific mortality in stage IA-IIB disease in Non-Nordic immigrants. In model adjusted for age a similar difference was observed for stage IIIA NSCLC. In advanced stage IIIB-IV lung cancer, there were no significant differences in mortality between native born and immigrant patients. (Table 4).

**Table 4:** Overall and cause specific mortality.

		Overall mortality						Cause-specific mortality					
	n	HR	95%y CI	aHR*	95% CI	aHR**	95% CI	HR	95% CI	aHR*	95% CI	aHR**	95% CI
**Stage IA-IIB, WHO PS 0–2**	** **	** **	** **	** **	** **	** **	** **	** **	** **	** **	** **	** **	** **
Sweden	7801	1.00	reference	1.00	reference	1.00	reference	1.00	reference	1.00	reference	1.00	reference
Nordic	702	1.19	1.07–1.32	1.24	1.12–1.37	1.27	1.14–1.41	1.21	1.07–1.37	1.24	1.10–1.41	1.31	1.15–1.48
Non-Nordic	851	0.81	0.73–0.90	0.94	0.85–1.05	0.88	0.79–0.98	0.80	0.70–0.91	0.90	0.79–1.02	0.86	0.75–0.98
**Stage IIIA, WHO PS 0–2**	** **												
Sweden	2764	1.00	reference	1.00	reference	1.00	reference	1.00	reference	1.00	reference	1.00	reference
Nordic	251	1.12	0.96–1.29	1.16	1.01–1.35	N/A	N/A	1.12	0.96–1.31	1.16	0.99–1.36	N/A	N/A
Non-Nordic	344	0.75	0.65–0.86	0.85	0.74–0.98	N/A	N/A	0.75	0.64–0.86	0.83	0.72–0.97	N/A	N/A
**Stage IIIB-IV, WHO PS 0–2**	** **												
Sweden	16104	1.00	reference	1.00	reference	1.00	reference	1.00	reference	1.00	reference	1.00	reference
Nordic	1362	1.03	0.97–1.09	1.03	0.97–1.09	0.99	0.93–1.05	1.03	0.97–1.09	1.03	0.97–1.09	0.99	0.93–1.05
Non-Nordic	1783	0.92	0.88–0.97	0.97	0.92–1.03	0.97	0.92–1.02	0.92	0.87–0.97	0.95	0.90–1.01	0.95	0.90–1.01

Overall and cause specific mortality in patients with performance status 0–2 diagnosed with non-small-cell lung cancer in Sweden 2002–2016 by stage at diagnosis and geographic region of birth.

* hazard ratio adjusted for age at diagnosis

** hazard ratio adjusted for age at diagnosis, level of education, CCI, stage at diagnosis, gender, year of diagnosis, performance status, smoking history and histology

N/A: Due to low number of patients, adjustments beyond age were not possible in Stage IIIA, WHO PS 0–2

Abbreviations: WHO PS: World Health Organization Performance Score.

In contrast, both overall and cause-specific survival was lower in Nordic immigrants with stage IA-IIIA disease compared to Swedish born patients (Fig 1). Following adjustments, these findings remained significant in stage IA-IIB, but not in more advanced disease.

## 4. Discussion

### 4.1 Summary of results

In this retrospective nationwide cohort study of patients with NSCLC immigrants from Non-Nordic countries were younger at diagnosis, had a higher educational level and a better performance status compared to Nordic immigrants and Swedish born patients reflecting the presence of a ‘healthy migrant effect’. We found only minor differences in patterns of care that were attenuated after adjustment for age and other potential confounding factors. If anything, immigrants from Non-Nordic countries were more often offered PET, were discussed in a multidisciplinary team setting and had lower mortality in early stage disease. Taken together, we found no evidence that patients with an immigrant background are disadvantaged when management decisions are made.

### 4.2 Comparisons to previous studies

In Sweden, almost one fifth of the total population of 10.3 million inhabitants had an immigrant background in 2019, i.e. born outside of Sweden [15]. Since 1955, Sweden has a tax-financed universal health care system aiming to provide all citizens access to health care at low out-of-pocket cost. Immigrants with a permanent residence permit obtain the same rights to health care as native Swedes and are offered free of charge interpreter services.

Our observation of special characteristics of the immigrant population, i.e. the healthy migrant effect, corroborate findings in previous studies [9–11, 23]. Younger age at time of a lung cancer diagnosis in immigrants may reflect differences in lifetime smoking history, lifestyle and environmental and occupational exposures [7]. Ethnic differences in genomic features, for example EGFR-expression in European and East Asian populations, and the interaction between genes and other factors may influence not only age at onset, but also outcome [11, 24, 25].

Our findings broadly confirm results of earlier studies conducted in Denmark, Sweden and Norway that had a primary focus on incidence and survival [7, 23, 26–28]. Compared to the native Norwegian population, Hjerkind et al found a higher incidence of lung cancer in immigrants from other Nordic countries and Eastern Europe [23]. Similar to our findings, the Norwegian study found no evidence of more advanced stage disease in the immigrant population [29]. To the best of our knowledge, only one previous study has compared patterns of lung cancer management between native born and immigrants [7]. Similar to the results of that study, we found that treatment according to guidelines was readily offered also to patients with an immigrant background. A high use of PET-DT in Non-Nordic immigrants may reflect that a majority reside in urban areas with large hospitals where this diagnostic modality is readily available. Koyi et al and Norredam et al found no significant differences in lung cancer mortality between native born and immigrant patients in Sweden and Denmark [7, 28]. Similar to our findings, a lower lung cancer mortality in immigrants has been reported from Norway [27]. Based on the results of the adjusted analyses in our study, we conclude that the better prognosis in Non-Nordic immigrants most likely reflect younger age at diagnosis. We have no clear explanation of our findings of poorer outcomes in Nordic immigrants with early-stage lung cancer. One possible explanation is a predominance of Finnish immigrants that have documented high mortality rates associated with socioeconomic factors, alcohol-related morbidity and cardiovascular disease. [30, 31].

### 4.3 Strengths and limitations

Strengths of our study included the use of data available in high quality population-based registers containing detailed individual level information on not only patient and tumor characteristics, but also management and outcomes. Because of the nationwide setting, the size of the dataset and the high completeness of the National Lung Cancer Register, selection bias was not an issue.

Limitations included the absence of detailed information on genetics, language skills, lifestyle factors, health awareness, health care seeking behavior and ability to navigate the health care system. Data on region of origin, but not specific country of origin were made available from Statistics Sweden. Due to small numbers, further subgrouping of Non-Nordic immigrants based on geographic region of origin was not possible. Information on smoking history was self-reported and is therefore subject to misclassification due to misreporting or recall bias. The assessment of comorbidity burden was based on in- and outpatient hospital care records while diagnoses made in primary health care setting was unavailable. For this reason, the comorbidity burden is likely to have been underestimated. Furthermore, no information was available to correctly characterize patients that died shortly after diagnosis or start of treatment. We were also unable to account for unregistered return of immigrants to their country of origin after a lung cancer diagnosis resulting in loss of follow-up and overestimation of survival (i.e. the so called salmon effect) [27].

## 5. Conclusion

We found only minor differences in patterns of management and outcomes between immigrant and Swedish born patients with lung cancer. We conclude that lung cancer care is offered on equal terms, regardless of patient background. If anything, outcomes were better in Non-Nordic immigrants with early stage disease.

## Supporting information

S1 Table The likelihood of surgery in patients diagnosed with non-small cell lung cancer in Sweden 2002–2016 with stage IA-IIB and performance status 0–2 by geographic region of birth. (DOCX)

S2 Table The likelihood of stereotactic radiotherapy (SBRT) in patients diagnosed with non-small cell lung cancer in Sweden 2002–2016 with stage IA-IIB and performance status 0–2 by geographic region of birth. (DOCX)

S3 Table The likelihood of surgery in patients diagnosed with non-small cell lung cancer in Sweden 2002–2016 with stage IIIA and performance status 0–2 by geographic region of birth. (DOCX)

S4 Table The likelihood of concurrent chemoradiotherapy in patients diagnosed with non-small cell lung cancer in Sweden 2002–2016 with stage IIIA and performance status 0–2 by geographic region of birth. (DOCX)
