# Supplementary material for: Patterns of care and outcomes in immigrants with non-small cell lung cancer. A population-based study (Sweden)
Source: PLoS One. 2022 Dec 15;17(12):e0278706. doi: 10.1371/journal.pone.0278706 (PMC9754210; doi:10.1371/journal.pone.0278706)
Supplement: S1 Table — (DOCX) [file pone.0278706.s001.docx]

| **S1 Table.** The likelihood of surgery in patients diagnosed with non-small cell lung cancer in Sweden 2002-2016 with stage IA-IIB and performance status 0-2 by geographic region of birth. | | | | | | | |
| --- | --- | --- | --- | --- | --- | --- | --- |
|  |  |  |  |  |  |  |  |
|  |  |  |  |  |  |  |  |
|  | **Likelihood of Surgery** | | | | | | |
|  | **OR** | **95% CI** |  | **aOR*** | **95% CI** | **aOR**** | **95% CI** |
|  |  |  |  |  |  |  |  |
| **Region of birth** |  |  |  |  |  |  |  |
| Sweden | 1.00 | reference |  | 1.00 | reference | 1.00 | reference |
| Nordic | 0.84 | 0.71-0.99 |  | 0.73 | 0.60-0.87 | 0.72 | 0.58-0.89 |
| Non-Nordic | 1.29 | 1.09-1.52 |  | 0.85 | 0.71-1.02 | 0.83 | 0.67-1.03 |
|  |  |  |  |  |  |  |  |
| * odds ratio adjusted for age at diagnosis | | | | | |  |  |
| ** odds ratio adjusted for level of education, CCI, age at diagnosis, stage at diagnosis, gender, year of diagnosis, performance status, smoking history and histology | | | | | | | |
|  |  |  |  |  |  |  |  |
