# Supplementary material for: Patterns of care and outcomes in immigrants with non-small cell lung cancer. A population-based study (Sweden)
Source: PLoS One. 2022 Dec 15;17(12):e0278706. doi: 10.1371/journal.pone.0278706 (PMC9754210; doi:10.1371/journal.pone.0278706)
Supplement: S2 Table — (DOCX) [file pone.0278706.s002.docx]

| **S2 Table.** The likelihood of stereotactic radiotherapy (SBRT) in patients diagnosed with non-small cell lung cancer in Sweden 2002-2016 with stage IA-IIB and performance status 0-2 by geographic region of birth. | | | | | | | |
| --- | --- | --- | --- | --- | --- | --- | --- |
|  |  |  |  |  |  |  |  |
|  |  |  |  |  |  |  |  |
|  | **Likelihood of SBRT** | | | | | | |
|  | **OR** | **95% CI** |  | **aOR*** | **95% CI** | **aOR**** | **95% CI** |
|  |  |  |  |  |  |  |  |
| **Region of birth** |  |  |  |  |  |  |  |
| Sweden | 1.00 | reference |  | 1.00 | reference | 1.00 | reference |
| Nordic | 1.27 | 0.94-1.72 |  | 1.38 | 1.01-1.89 | 1.36 | 0.94-1.96 |
| Non-Nordic | 1.06 | 0.79-1.43 |  | 1.08 | 1.07-1.09 | 1.53 | 1.06-2.21 |
|  |  |  |  |  |  |  |  |
| * odds ratio adjusted for age at diagnosis | | | | | |  |  |

** odds ratio adjusted for level of education, CCI, age at diagnosis, stage at diagnosis, gender, year of diagnosis, performance status, smoking history and histology
