# Supplementary material for: Patterns of care and outcomes in immigrants with non-small cell lung cancer. A population-based study (Sweden)
Source: PLoS One. 2022 Dec 15;17(12):e0278706. doi: 10.1371/journal.pone.0278706 (PMC9754210; doi:10.1371/journal.pone.0278706)
Supplement: S3 Table — (DOCX) [file pone.0278706.s003.docx]

| \| **S3 Table.** The likelihood of surgery in patients diagnosed with non-small cell lung cancer in Sweden 2002-2016 with stage IIIA and performance status 0-2 by geographic region of birth. \| \| \| \| \| \| \| \| \| --- \| --- \| --- \| --- \| --- \| --- \| --- \| --- \| \| \| \|  \| **Likelihood of Surgery** \| \| \| \| \| \| \| \|  \| **OR** \| **95% CI** \|  \| **aOR*** \| **95% CI** \| **aOR**** \| **95% CI** \| \|  \|  \|  \|  \|  \|  \|  \|  \| \| **Region of birth** \|  \|  \|  \|  \|  \|  \|  \| \| Sweden \| 1.00 \| reference \|  \| 1.00 \| reference \| 1.00 \| reference \| \| Nordic \| 0.85 \| 0.59-1.22 \|  \| 0.76 \| 0.53-1.09 \| N/A \| N/A \| \| Non-Nordic \| 1.53 \| 1.17-1.99 \|  \| 1.17 \| 0.89-1.54 \| N/A \| N/A \| \|  \|  \|  \|  \|  \|  \|  \|  \| \| * odds ratio adjusted for age at diagnosis \| \| \| \| \| \|  \|  \| \| ** odds ratio adjusted for level of education, CCI, age at diagnosis, stage at diagnosis, gender, year of diagnosis, performance status, smoking history and histology \| \| \| \| \| \| \| \| \| |
| --- | --- | --- | --- | --- | --- | --- | --- | --- | --- | --- | --- | --- | --- | --- | --- | --- | --- | --- | --- | --- | --- | --- | --- | --- | --- | --- | --- | --- | --- | --- | --- | --- | --- | --- | --- | --- | --- | --- | --- | --- | --- | --- | --- | --- | --- | --- | --- | --- | --- | --- | --- | --- | --- | --- | --- | --- | --- | --- | --- | --- | --- | --- | --- | --- | --- | --- | --- | --- | --- | --- | --- | --- | --- | --- | --- | --- | --- | --- | --- | --- | --- | --- | --- | --- | --- | --- | --- | --- |
|  |
